# Diet quality and food security amongst Indigenous children in Canada: facing the legacy of decades of dispossession and governmental neglect

**DOI:** 10.1017/S1368980021003487

**Published:** 2022-01

**Authors:** Malek Batal

**Affiliations:** Canada Research Chair in Nutrition and Health Inequalities, Nutrition Department, Faculty of Medicine, Université de Montréal, Centre for Public Health Research (CReSP) of the Université de Montréal and the CIUSS du Centre-Sud-de-l’Île-de-Montréal, TRANSNUT, WHO – Collaborating Centre on Nutrition Changes and Development, CP 6128 Succ. Centre-Ville, Montréal, QC H3C 3J7, Canada

Canada is a prosperous country and member of the G7, with an enviable track record in public safety nets, including child support and public medical care^(1)^. However, Indigenous peoples who form 5 % of the population of the country and include First Nations, Metis, and Inuit, still face, as a group, challenges not faced by the settler majority. They continue to be confronted with systematically worse determinants of health including housing, job prospects and income, and educational attainment than the rest of the population^(2)^, a legacy of a paternalistic system that systematically provided inferior services and care^(3)^. Consequently, Indigenous children and adults face a higher prevalence of obesity and nutrition and lifestyle-related chronic disease and live more frequently in food-insecure households^(4–7)^. In addition, rates of injury and violent deaths, suicide, and infectious diseases continue to be higher for this population^(8–11)^. Meanwhile, communities are striving to build on their strengths, reverse the unfavourable trends and build resilience through health and wellness programming that aim to revitalise the traditional food systems, as well as through the transmission of culturally foundational knowledge about the land, and the skills needed for the continued access to traditional food through hunting, fishing, gathering, and cultivating^(12–15)^. These programmes are often based on holistic Indigenous worldviews that consider human health as a continuation of the health of the ecosystem and all living and non-living things^(16,17)^.

In the recently published results of the First Nations Food, Nutrition and Environment Study (FNFNES.ca) spanning 10 years (2008–2018) conducted with 92 First Nations communities with a large sample of 6500 adult participants from territorial entities still officially named ‘reserves’ across Canada^(18)^, it was found that the diet of First Nations was often inadequate, particularly since many barriers (government regulations, industrial activities like mining and forestry, dams for the generation of hydroelectricity, lack of time, erosion of traditional knowledge about the land and traditional harvesting activities, lack of money for hunting/fishing equipment, etc.) prevent community members from accessing their ancestral territory, thus affecting food supply and limiting their enjoyment of traditional food, which is of much greater nutritional quality than the generally available market food, particularly in remote communities^(19,20)^. In fact, on days when traditional food was consumed, intakes of key nutrients were greatly improved compared to days without any traditional food in the diet^(19)^. It was also found that obesity and diabetes were very high, at 50 % and 25 %, respectively, double and three times higher than in the general Canadian population^(7)^. Most disturbingly, food insecurity (the inability to access food because of lack of financial means) was, at 48 %, four times higher in this representative sample of First Nations living on reserve in the ten Canadian provinces than the 12 % rate amongst the Canadian population, which is still considered high for a rich country like Canada^(6)^. Food insecurity is also experienced at a higher rate in adults living in households with children, with parents reporting lower food insecurity for the children themselves, indicating an attempt at sheltering the children from food insecurity^(6,21)^. Food security was measured using the Household Food Security Survey Module (HFSSM) with respondents categorised as living in households that were food secure, marginally food insecure, moderately food insecure, or severely food insecure. The HFSSM comprises eighteen questions about the food security situation over the previous 12 months with a section focused on children’s food security^(22)^.

While these results from First Nations adults have been recently published in a special issue of the Canadian Journal of Public Health^(23)^, little is known about the nutritional and food security situation for Indigenous children and youth until the publication in this issue of an important article comparing results for off-reserve Indigenous and non-Indigenous children and between 2004 and 2015 based on the Canadian Community Health Survey (CCHS) nutrition data. The article titled ‘Diet quality among Indigenous and non-Indigenous children and youth in Canada in 2004 and 2015: a repeated cross-sectional design’ explores changes in diet quality using the Healthy Eating Index (HEI), a score that measures diet quality in terms of adequacy and moderation by assessing the degree of adherence of the diet of surveyed individuals to the recommendations of the 2007 version of Canada’s Food Guide. The CCHS is a regularly conducted population survey with only the nutrition component, which includes a 24-h recall questionnaire repeated with 20 % of the sample, only administered twice in the recent past. Results from the survey are generalisable to the Canadian population thanks to its sampling framework. It is possible to identify Indigenous participants in the sample based on self-reports of Indigenous identity. However, Inuit participants are so few in CCHS that no conclusions could be drawn from the survey for Inuit living outside of the Inuit villages. It is important to note that national surveys in Canada, such as the CCHS, exclude First Nations and Metis living on reserves or communities and Inuit living in the north as well as Canadians living in remote communities^(24,25)^.

Using data from the 24-h recalls, the article proposes a positive reading of the nutritional situation of children and youth aged 2–17 years, pointing to a generally improved HEI for both Indigenous and non-Indigenous children and youth between 2004 and 2015 and suggesting that these improvements, which are due largely to a reduced contribution of the ‘other’ food group in the diet (often snacks of low nutritional quality), are facilitated by the multiplication of food school programmes and interventions aimed at reducing consumption of junk food in this population. However, this improvement did not seem to be visible with Indigenous girls, indicating the need for a thorough investigation of gender dynamics in food distribution and consumption in that population.

While this is an encouraging trend, the article also notes the persistence of the great disparity between Indigenous and non-Indigenous children and youth in terms of food security as measured by the USDA Household Food Security Survey Module. In fact, food insecurity was three times higher in Indigenous than non-Indigenous children and youth in both 2004 and 2015, with only modest improvement in Indigenous food insecurity rates over the 11 years which does not consider the marginally food insecure category (from 32·8 % to 28·3 %). While the exploration of this disparity was beyond the scope of the article, it would have been useful to have this reality be made more prominent and included in the title. Food insecurity is a major determinant of diet quality (as seen in the article results) and is also associated with both mental and physical ill health and a reduced ability to manage disease^(26–28)^. As Canada grapples with its colonialist past’s impact on health^(9,29)^ and Indigenous people are forced to deal with historic traumas recently evidenced by the gruesome discoveries of unmarked children’s burial sites close to some of the infamous residential schools^(30)^, the Indigenous population continues to also pay the price of continuing discrimination with suboptimal health, social, and economic outcomes^(31)^.

Improvements in the diet quality of off-reserve Indigenous children and youth are welcome, but the continuing very high levels of food insecurity are troubling. The UN Convention on the Rights of the Child stipulates that every child has the right to adequate housing and adequate nutritious foods and clean drinking water^(32)^. It is inexplicable that these basic human rights are still denied to a large number of Indigenous children in a wealthy country like Canada.

The CCHS data and the article by Riediger *et al.* allow us a glimpse into the nutritional and food security reality of Indigenous children and youth who live off-reserve. However, the situation on-reserve is very different because of a myriad of differing political, economic, and physical attributes in reserves compared to elsewhere in Canada. A new study modelled after the FNFNES but dedicated to understanding the nutrition and environmental situation of Indigenous children and youth living on-reserve is underway. The 10-year Food, Environment, Health and Nutrition of First Nations Children and Youth (FEHNCY.ca) study promises to draw a representative picture of the situation on-reserve and will allow comparisons with the general Canadian population as well as with off-reserve Indigenous children and youth. This should deepen our understanding and provide insight into changes needed and, we dare hope, track improvements as nutritional, health, education, housing, and other gaps narrow with better resources, greater self-determination, and programmes that prioritise access to traditional foods.
